# Broad complex rhythm with a salty taste

**DOI:** 10.1007/s12471-017-0950-y

**Published:** 2017-01-20

**Authors:** M. Boulaksil, C. L. Meuwese, R. Evertz, M. G. M. Kolff-Kamphuis

**Affiliations:** 1 Department of Cardiology, Jeroen Bosch Hospital, ’s-Hertogenbosch, The Netherlands; 2 Department of Cardiology, Radboud University Medical Center, Nijmegen, The Netherlands; 3 Department of Cardiology, University Medical Center Utrecht, Utrecht, The Netherlands

A 65-year-old female patient with a medical history of stroke and paroxysmal atrial fibrillation presented to the neurologist at the emergency department of our hospital because of progressive weakness, dyspnoea, and fatigue. Her fluid intake had been minimal because of her malaise. She had no history of syncope. She used the following medications: warfarin, flecainide, and metoprolol. Initial physical examination showed a blood pressure of 105/65 mmHg, a heart rate of 90/min, and no fever. She was clinically mildly decompensated: she had bilateral pulmonary rales and ankle oedema. A CT scan of the brain did not show any significant abnormalities. Laboratory results showed: creatinine 108 µmol/l; MDRD-GFR 44 ml/min; sodium 137 mmol/l; potassium 5.3 mmol/l; calcium 2.2 mmol/l; and magnesium 0.87 mmol/l.

Fig. 1 shows the initial ECG on admission. After taking her daily medication at the emergency department, the ECG evolved to Fig. 2. Eventually, she was admitted to the cardiac care unit.

**Fig. 1:**
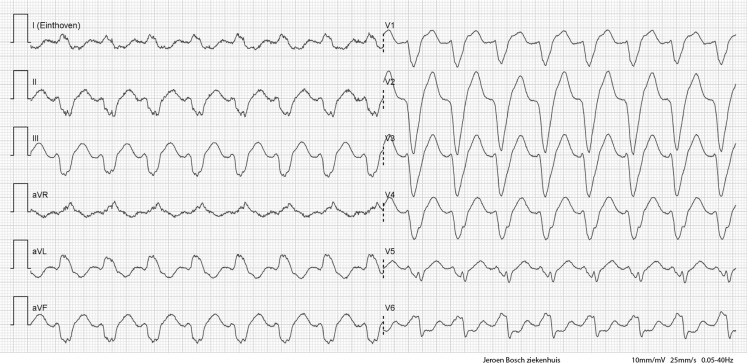
ECG upon arrival to the emergency department

**Fig. 2:**
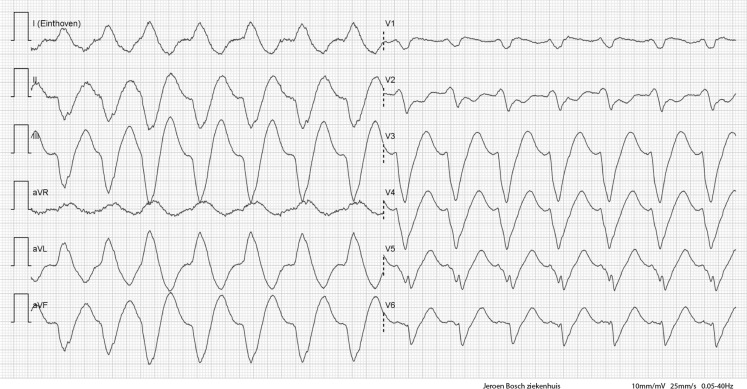
Follow-up ECG during stay at the emergency department

What is your (differential) diagnosis?

## Answer

You will find the answer elsewhere in this issue.

